# Quorum Sensing by Monocyte-Derived Populations

**DOI:** 10.3389/fimmu.2019.02140

**Published:** 2019-09-11

**Authors:** Jérémy Postat, Philippe Bousso

**Affiliations:** ^1^Dynamics of Immune Responses Unit, Institut Pasteur, INSERM U1223, Paris, France; ^2^Sorbonne Paris Cité, Cellule Pasteur, University Paris Diderot, Paris, France

**Keywords:** monocyte, monocyte-derived cell, quorum sensing (QS), macrophage, nitric oxide, metabolism

## Abstract

Quorum sensing is a type of cellular communication that was first described in bacteria, consisting of gene expression regulation in response to changes in cell-population density. Bacteria synthesize and secrete diffusive molecules called autoinducers, which concentration varies accordingly with cell density and can be detected by the producing cells themselves. Once autoinducer concentration reaches a critical threshold, all bacteria within the autoinducer-rich environment react by modifying their genetic expression and adopt a coordinated behavior (e.g., biofilm formation, virulence factor expression, or swarming motility). Recent advances highlight the possibility that such type of communication is not restricted to bacteria, but can exist among other cell types, including immune cells and more specifically monocyte-derived cells (1). For such cells, quorum sensing mechanisms may not only regulate their population size and synchronize their behavior at homeostasis but also alter their activity and function in unexpected ways during immune reactions. Although the nature of immune autoinducers and cellular mechanisms remains to be fully characterized, quorum sensing mechanisms in the immune system challenge our traditional conception of immune cell interactions and likely represent an important mode of communication at homeostasis or during an immune response. In this mini-review, we briefly present the prototypic features of quorum sensing in bacteria and discuss the existing evidence for quorum sensing within the immune system. Mainly, we review quorum sensing mechanisms among monocyte-derived cells, such as the regulation of inflammation by the density of monocyte-derived cells that produce nitric oxide and discuss the relevance of such models in the context of immune-related pathologies.

## Quorum Sensing: From the Bacterial World and Beyond

Living cell-based systems, such as multicellular organisms or bacterial biofilms, are very complex collections of biological components (cells) continually interacting with each other. They are organized as coordinated functional communities, which integrity relies on an efficient organization, and therefore, communication. Cells communicate with each other by various mechanisms either dependent on cell contact (e.g., juxtacrine signaling, membrane nanotubes) or dependent on diffusive material (e.g., diffusive signaling molecules, exosomes). Different modes of contact-independent signaling, including autocrine, paracrine, and endocrine signaling, were extensively characterized in the past using mammalian systems (2, 3). Autocrine signaling occurs when the same cell simultaneously produces and responds to a signaling molecule that stays confined to the cell vicinity, with the help of high-affinity receptors. By contrast, paracrine signaling happens when the responding cell is located at a relatively short distance from the cell producing the signal, and often of a different cell type. Lastly, endocrine signaling is defined when the signaling and the target cells are located at distant sites, the signaling molecule traveling by the bloodstream.

**Figure 1:**
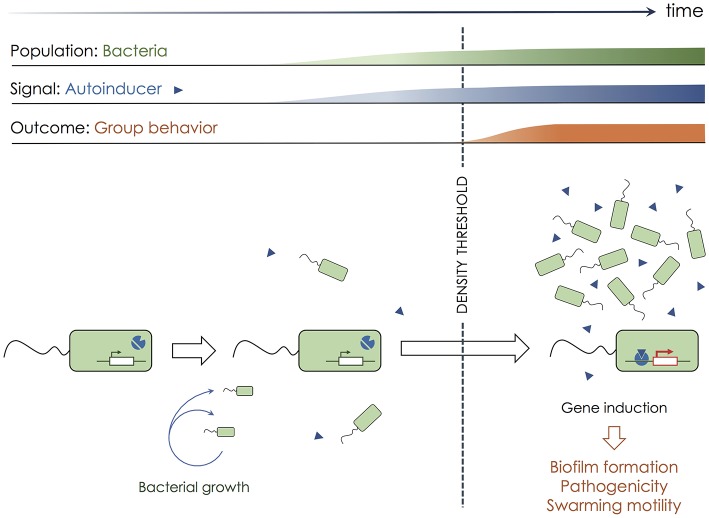
Quorum sensing in bacteria. To communicate and synchronize their behavior, bacteria (green rectangles) use quorum sensing. Each bacterium produces a low quantity of a membrane-diffusive molecule called autoinducer (blue triangles), which biological activity is absent at low concentration. Bacterial growth over time increases cell density together with the concentration of the autoinducer in the extracellular environment. Once a sufficient number of bacteria have accumulated, hence reaching a sufficient density, the autoinducer concentration is high enough to initiate biological alterations. In bacteria, the autoinducer often triggers a switch in genetic expression, after binding to transcriptional regulators. Such a genetic switch leads to the emergence of group behaviors such as biofilm formation, increased pathogenicity, or swarming motility.

Over 50 years ago, the most well-known bacterial mode of communication named quorum sensing was discovered in the luminous marine bacterial species *Vibrio fischeri* (4). Quorum sensing is a process of chemical communication that bacteria use to orchestrate group behaviors [(5–8); Figure 1]. In classic quorum sensing, each bacterium of the same type produces a membrane-diffusive signaling molecule called autoinducer, that can modify bacterial gene expression. The production of autoinducer by individual cells is too low to trigger any significant biological effect. When a threshold of cell density is reached (e.g., by continuous bacterial growth), the autoinducer reaches a sufficient concentration in the local environment to alter gene expression in all cells present in the area. The coordinated alteration of gene expression results in the emergence of group behaviors such as biofilm formation (9), virulence factor expression (10), or swarming motility (11). Therefore, quorum sensing mechanisms in bacteria initiate specific responses only when a sufficient cell density is reached, and rely on a diffusive molecule that acts as a surrogate for cell density (autoinducer). Quorum sensing mechanisms provide the possibility for spatiotemporal regulation of collections of cells, and for the emergence of specific behavior that would be unproductive when undertaken by a single isolated bacterium.

It is only recently that several immune regulatory processes similar to quorum sensing mechanisms have been revealed in the mammalian immune system. Notably, such processes were shown to be triggered locally only when a sufficient number of cells were reached and lead to population-level effects. Also, these mechanisms involve diffusive signaling molecules such as cytokines and chemokines, that resemble autoinducers in that they might be secreted in a reasonably low amount by single cells. Additionally, quorum sensing regulation may occur by mechanisms not only modifying gene expression, but also altering cellular metabolism.

In this mini-review, we will discuss the existence and biological relevance of quorum sensing mechanisms by immune cells at homeostasis and during inflammation, with a particular focus on monocyte-derived cells. We propose that quorum sensing mechanisms are integral to the immune system and that malfunction of such regulatory pathways may lead to uncontrolled monocyte-derived cell accumulation and activation, leading to excessive immune responses and the development of immunopathologies. We will not address the impact of bacterial quorum sensing molecules on monocyte activity, but rather extend the theory originally described in bacteria to the monocyte-derived cells.

## Mechanism of Quorum Sensing in Monocyte-Derived Cells

### Monocyte-Derived Cell Homeostasis

In mammalian species, the concept of homeostasis establishes that most tissues and organs are made of different cell types whose numbers remain constant at steady-state. Accordingly, monocyte-derived cells such as monocyte-derived macrophages can maintain their cellular density in different organs under physiological conditions (12, 13). Such maintenance is achieved in the different tissues by compensating cell death either by a continuous input of circulating monocytes or by a constant self-renewal of tissue-resident macrophages as for Langerhans cells in the murine epidermis (12, 14–16). However, most of the mechanisms governing monocyte-derived cell homeostasis are still poorly understood. Recently, Antonioli et al. suggested that macrophage homeostasis is controlled under physiological conditions by a quorum sensing mechanism (1, 17). They propose a central role for Colony-Stimulating Factor 1 (CSF1) as the autoinducer for macrophages, as it controls their survival and proliferation at steady-state (18–20). Macrophage density would be controlled by two factors: CSF1 production by stromal cells and its consumption by the entire population. Accordingly, a two-cell circuit-based model between Platelet-Derived Growth Factor (PDGF)-producing macrophages and CSF1-producing fibroblasts was shown to have sufficient stability and robustness to perturbation to allow macrophage/stromal cell homeostasis (21). Further studies will provide new insight regarding the relevance of this model *in vivo* and its potential role not only at steady-state but also during ongoing immune responses. While this particular mechanism somewhat differs from classic microbial quorum sensing in that the autoinducer is produced by stromal cells (which are not the responding cells), it still aims at controlling the macrophage pool size and may be best defined as an indirect quorum sensing mechanism. Finally, the idea that cell density could also affect macrophage behavior at homeostasis has been less investigated. In one study using a model of cultured human monocytes, it was established that the secretion of chondroitin sulfate proteoglycan (structural component of human tissues) by these cells was dependent on their cell density (22).

### Quorum Sensing During Immune Reactions

Other mechanisms controlling the size and activity of monocyte-derived cell populations were recently described during immune reactions. For instance, human macrophages infected by the intracellular pathogen *Mycobacterium tuberculosis* restrict bacterial growth more efficiently when cultivated at high density (23). The authors proposed that such finding is compatible with the possibility that high-density cultures release factors that can affect cell behavior [in that case, bacterial behavior]. More recently, TNF-α and IL-10 have been identified as the primary soluble mediators positively and negatively regulating macrophage function, capable of mediating cytokine production in groups vs. in single cells (1). Additionally, it was proposed that the regeneration of hair follicles in response to patterned hair plucking is regulated by a quorum sensing mechanism involving macrophages (24). The authors uncovered a two-step mechanism where CCL2 released from damaged hairs leads to the recruitment of TNF-α-producing macrophages which accumulate and signal to both plucked and unplucked follicles to stimulate their regeneration.

**Figure 2:**
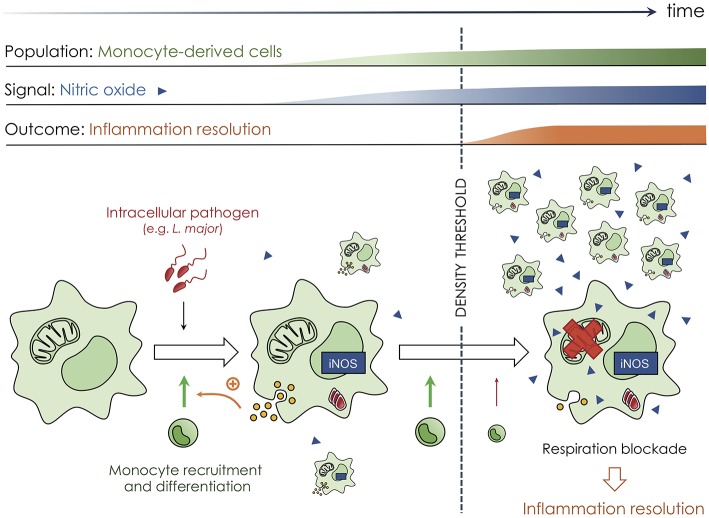
Quorum sensing among mononuclear phagocytes at the site of infection by intracellular pathogens. Mononuclear phagocytes are endowed with a quorum sensing mechanism during the immune reaction against *Leishmania major* parasites. Local skin infection with this intracellular pathogen elicits inflammation and the recruitment of innate immune cells from the blood, including monocytes (small round green cells) that differentiate into mononuclear phagocytes (large rough green cells) at the site of the immune reaction. Such cells sustain monocyte infiltration and differentiation by secreting cytokine and chemokine (yellow circles) but also produce nitric oxide (blue triangles, NO) that diffuses within the microenvironment and helps fight the infection. Such mechanism increases mononuclear phagocyte number at the site of infection during the early phases of the response, allowing for local control of the pathogen load. Once a sufficient number of mononuclear phagocytes have accumulated, NO starts to repress cellular respiration (red cross on the mitochondria), dampening the cellular ATP:ADP ratio and ultimately limiting cytokine and chemokine secretion that is needed for immune cell recruitment. The mechanism relies on NO that diffuses and acts on every mononuclear phagocyte, independently of their iNOS expression, and only exists when a sufficient number of cells have accumulated. Therefore, NO acts as an autoinducer for mononuclear phagocytes, limiting their recruitment and the development of an immunopathology but only when a sufficient number of cells have accumulated to control the infection efficiently.

More recently, a quorum sensing mechanism more similar to what described in bacteria was comprehensively described *in vivo* during the immune response against *Leishmania major* [(25); Figure 2]. Local infection with this intracellular parasite in murine skin triggers a massive recruitment of immune cells at the site of infection, including circulating monocytes. Recruited monocytes differentiate into mononuclear phagocytes that not only represent the main population of infected cells but also actively participate in controlling the local inflammatory reaction. Such an immune response can be detrimental to the host by inducing irreversible tissue damage if not adequately regulated and terminated on time (26, 27). Controlling mononuclear phagocyte recruitment, activity, and clearance is therefore essential to resolve inflammation concomitantly to parasite elimination. During cutaneous leishmaniasis, recruited mononuclear phagocytes secrete nitric oxide (NO) that acts to adjust and limit the overall inflammation intensity. NO suppresses mononuclear phagocyte accumulation, as well as cytokine and chemokine production by blocking cellular respiration and decreasing the ATP:ADP ratio. Importantly, such mechanism only exists when a sufficient number of mononuclear phagocytes have accumulated at the site of infection (more than 5,000 cells per mm^3^) to produce a high quantity of NO in a collective manner (Figure 2). NO acts thereafter by diffusing and targeting all mononuclear phagocytes irrespectively of their intrinsic iNOS expression. In this mechanism, the mitochondria, and most probably the cytochrome c oxidase, represents the target of NO (28, 29). Therefore, mononuclear phagocytes not only produce NO but are also regulated in number and activity by this diffusive molecule, establishing a quorum sensing mechanism for the control of the inflammatory reaction, with NO acting as the autoinducer. Furthermore, it is very interesting to note that such mechanism relies on the modification of cellular metabolism. That shows, in contrast to the current paradigm in bacteria, that quorum sensing mechanisms do not necessarily rely on genetic alteration. Targeting cellular metabolism compared to genetic expression could have several advantages, including the possibility to alter cell activity within a very short period of time and in a rapidly reversible manner (25). In addition to dampening oxidative phosphorylation, NO was also shown to accelerate myeloid cell death (30, 31), a phenomenon that could participate in inflammation resolution (32). We propose that this NO-based quorum sensing mechanism is integral to the biology of inflammatory mononuclear phagocytes and could certainly operate in other models of infections or during cancer development. We additionally propose that malfunction of quorum sensing mechanisms may lead to uncontrolled mononuclear phagocyte accumulation and activation, leading to non-resolving inflammation and therefore immunopathology development (26).

Furthermore, other quorum sensing mechanisms are about to be elucidated during immune reactions. Indeed, using a droplet-based microfluidic approach, Wimmers et al. showed that during human pDCs activation, the fraction of IFN-α-expressing cells at early time points of activation is dependent on their initial cell density. Using microtiter plates, they measured that more than 10^3^ pDCs per well should be stimulated together to have a significant percentage of IFN-α-producing cells following activation (33). That suggests that the magnitude of dendritic cell activity significantly increases above a threshold number of cells and that these cells are probably endowed with quorum sensing capability. Similarly, it was recently showed using mathematical modeling that macrophage activation is most probably bimodal, with a proportion of highly activated cells increasing with cell density. Because only a fraction of the population becomes activated, the authors preferably describe such phenomenon as quorum licensing rather than quorum sensing (34). Such studies and early work on human macrophages establish the potential relevance of quorum sensing mechanisms for the control of immune reaction in humans.

### Benefits Over and in Conjunction With Autocrine and Paracrine Signaling

Immune cell communication by quorum sensing mechanisms provides many benefits over autocrine or paracrine signaling. Quorum sensing mechanisms can significantly differ from paracrine signaling, a distinction that is not systematically present in the literature. To distinguish the two communication modes, further studies will have to investigate whether the diffusive molecule has any biological effect when the producing cell is present at low density. Indeed, the absence of biological effect of the signaling molecule (autoinducer) when its producer is at too low density is a cornerstone of quorum sensing. In other words, if a diffusive molecule biologically acts when the producer cell is at a negligible density, the communication occurs through paracrine signaling.

A first advantage of quorum sensing is the regulation of the cell number *per se* in tissues during inflammation (35). For instance, a high T cell density is needed for their terminal differentiation into cytotoxic T cells (36). By contrast, an excessive number of cells involved in an immune response can trigger immunopathology (25). Quorum sensing mechanisms may therefore locally adjust the cell density to promote or stop the immune reaction. Next, compared to autocrine signaling, the use of a diffusive mediator (the autoinducer) that acts simultaneously on numerous cells offers a way to reduce the level of heterogeneity between cells, and therefore coordinate their behavior in their complex microenvironment. For instance, DC populations can synchronize their behavior at late time points of activation provided that a small fraction of them secrete type I interferon rapidly after stimulation (37). Also, compared to paracrine signaling, quorum sensing mechanisms have the potential to temporally and locally adjust cell density and inflammation intensity without the need for external cues or regulatory cells. Paracrine signaling would not allow such self-adjustment to exist because it implies at least two cell types: the producing and the target cells. In a way, quorum sensing mechanisms can be considered as a form of paracrine signaling that depends on the cell population density but in which cells produce both a signaling molecule and its receptor, as in autocrine signaling (38).

Nonetheless, these different types of communication do not seem mutually exclusive. For instance, during the immune reaction against the parasite *L. major* in murine skin, a two-wave immune cell regulation occurs (39). First, a wave of IFN-γ secreted by activated CD4^+^ T cells spreads away from the site of antigen presentation and induces iNOS expression in numerous infected and bystander monocyte-derived cells, by a mechanism resembling paracrine signaling (40). Next, the subsequent collective production of NO allows for both parasite control (41) and regulation of the inflammation intensity at the tissue level by a metabolism-based quorum sensing mechanism (25). Thus, both paracrine signaling and quorum sensing can act in concert to spread a signal originating from a few discrete sites of cell activation to the level of the entire organ, while keeping the inflammatory reaction locally under tight control. Another example of coupling also exists for type I IFN during pDCs activation. In such an event, IFN-α stimulates its own production and alter cellular metabolism via an autocrine amplification loop (33, 42) but also regulates the fraction of IFN-α-producing cell most probably by a quorum sensing mechanism. Indeed, the fraction of activated pDCs is dependent on their cell density and relies on the diffusion of IFN-α that binds most probably IFNAR1 to mediates its biological effects (33, 43). Hence, combining quorum sensing with either autocrine or paracrine signaling appears to be essential to fine-tune immune cell activity.

## Quorum Sensing by Immune Cells: Perspectives

### Quorum Sensing by Other Immune Cell Types

While we focused on monocyte-derived cells, quorum sensing mechanisms have been described as well in other immune cells such as T and B cells.

In T cells, interleukin-2 (IL-2) was shown to be a significant autoinducer involved in a quorum sensing regulatory loop stabilizing T cell population density and phenotype (44–48). With the help of mathematical models and *in vitro* experiments, it was shown that a sufficient density of T_EFF_ cells is critical to reach a minimum threshold of IL-2 above which the phosphorylation of the signal transducer and activator of transcription (STAT) 5 is sustained to allow T cell proliferation (45). It was demonstrated using the same strategy that an excess of IL-2 above a maximal threshold leads to cell death instead of proliferation, participating in the regulation of T cell density to reach homeostasis (46). As well, it was recently evidenced that T cell density can directly modulate T cell differentiation toward T_EFF_ or T_CM_ by a quorum sensing mechanism relying on IL-2 and IL-6 as autoinducers (47). Additionally, during an immune challenge, T cells were shown to establish a negative feedback loop by capturing their cognate pMHC complexes from antigen-presenting cells and presenting them to antigen-experienced CD4^+^ T cells, thereby inhibiting their recruitment into the ongoing response (49). Finally, it has been proposed that T cell activation require a quorum of lymphocytes to happens (50).

Additionally, B cells were also shown to be endowed with a quorum sensing regulatory mechanism (51). After challenge, activated B cells secrete soluble immunoglobulin G (IgG) which concentration rises in the serum and is detected by the inhibitory receptor FcγRIIB, present on all B cells. At a sufficient concentration of IgG, the binding on this receptor is enough to trigger intracellular inhibitory pathways and prevent further IgM-secreting B cell activation. As a result, the number of IgM-secreting B cells is kept under control (51). So far, only IgG molecules were shown to act as autoinducer for B cells.

### Outstanding Questions and Perspectives

We envision that quorum sensing is integral to the regulation of immune responses. In this respect, future studies are critically needed to extend our understanding of this mode of communication. Several outstanding questions remain open: How autoinducer concentrations evolve in tissues during immune reactions? How can the physical tissue architecture and organ structure impact autoinducer diffusion, distribution, and access to the cells? How accurately can a cell population sense its own density based on an autoinducer concentration? Are there other unpredicted advantages of quorum sensing in immune cells? Do regulatory mechanisms for quorum sensing, such as quorum sensing quenching (52), can be triggered by invading pathogens or by the immune cells themselves? Can we target quorum sensing mechanisms in the context of immunotherapies (53). Further studies in both bacterial and mammalian systems will help answer these questions (54–57).

Given the complexity of the immune system and the vast number of quorum sensing mechanisms described in bacteria, we anticipate systems immunology to be essential in unraveling new quorum sensing mechanisms in immune cells (58). Mathematical modeling and computational simulations should help identify new quorum sensing mechanisms in immune cells, as it has been done for T cells (46, 59, 60) and more recently in macrophages (34). Furthermore, the development of new biological tools to measure autoinducer concentration and diffusion in complex tissue microenvironment are needed. For instance, reliable tools to map IL-2 or nitric oxide gradient *in vivo* would be of great help to better decipher quorum sensing mechanisms and characterize the effect of tissue architecture of its efficacy.

## Concluding Remarks

While quorum sensing is the norm in the bacteria world, it is only recently that similar mechanisms were shown to exist in the immune system, including in monocyte-derived cells. Quorum sensing mechanisms provide a way to regulate immune cell activity concurrently in a spatial and temporal manner complementary to what autocrine or paracrine signaling can achieve. They also favor the emergence of group behaviors and synchronized responses, two features decreasing the sensitivity of the system to external perturbations and therefore increasing its robustness. Alteration of immune quorum sensing mechanisms by impaired access or dysregulated response to the autoinducer may certainly trigger the emergence of immunopathology, as demonstrated in the context of the infection by the parasite *L. major*. Future studies are needed to extend the concept to other cell types and models to provide a better understanding of how this unique mode of communication integrates within the complexity of immune reactions.

## Author Contributions

All authors listed drafted, wrote, and edited the manuscript until the final version was approved and contributed to figures.

### Conflict of Interest Statement

The authors declare that the research was conducted in the absence of any commercial or financial relationships that could be construed as a potential conflict of interest.
